# The Roentgen Diagnosis of Diseases of the Gastro-Intestinal Tract*Read before Buffalo Academy of Medicine, April 12, 1916.

**Published:** 1917-01

**Authors:** Lester Levyn

**Affiliations:** Roentgenologist Erie County Hospital, Buffalo. Physician German Hospital Dispensary


### BUFFALO MEDICAL JOURNAL

Yearly Volume 72 JANUARY, 1917 Number 6

### ORIGINAL ARTICLES

The right is reserved to decline papers not dealing with practical medical and surgical subjects, and such as might offend or fail to interest readers. Contributors are solely responsible for opinions, methods of expression and revision of proof.

---

### The Roentgen Diagnosis of Diseases of the Gastro-Intestinal Tract.*

LESTER LEVYN, M. D.,

Roentgenologist Erie County Hospital, Buffalo.

Physician German Hospital Dispensary.

In introducting this paper I am taking the liberty of quoting the late Dr. Chas. Lester Leonard, "The importance of Roentgenology in the diagnosis of Gastro-Intestinal lesions has recently been emphasized by the prominence given to this subject in the programs of National Medical and Surgical Societies. Special reports have been made and entire sessions given over to their discussion. The progress in the past five years has influenced medical and surgical opinion until a Roentgen examination is considered a sine qua non before operation. The kindred sciences of anatomy, physiology, and pathology show the effect of their study in the living man by this method. Other methods of physical and chemical diagnosis are not displaced, but are amplified by the knowledge it affords. In fact the Roentgen method yields its best results when the patient referred for examination is accompanied by all the data which the older methods can secure."

Since Roentgen technique has improved so extensively during the past five years, this comparatively recent means of diagnosis has found a permanent place in modern medicine. It is of inestimable value as an aid in solving many difficult problems in the field of Gastro Enterology as in other branches of medicine. It remained for the X-ray to give us

---

*Read before Buffalo Academy of Medicine, April 12, 1916.

a correct interpretation of the size, shape, position, motility and character of peristalsis of the stomach and intestines in the living. After death or during the progress of an operation under general anaesthesia the stomach is entirely different than while performing its usual functions during life.

Several types of normal stomachs have been described by various investigators. Among these are the steer horn form, fish hook form and text book stomach. On the continent the two forms most generally recognized are those described by Rieder and Holznecht, known as the Rieder form and Holznecht form stomachs. The normal stomach in a stout person differs considerably from that of a thin emaciated subject, it being higher and occupying a more transverse position, due to the support of the abdominal fat, while in the very thin the stomach may be situated in the pelvis.

I shall not consume more time discussing the normal stomach, but will consider certain abnormal conditions with which we are daily confronted.

### GASTRIC CANCER

The Roentgen examination of the stomach has proved a decided adjunct in the diagnosis of early Gastric Cancer. Like all other methods, however, we must not consider it infallible. In early cancer before marked obstruction has occurred, we are frequently able to determine partial obstruction earlier than by any other methods. In some cases of beginning cancer of the stomach the Roentgen signs simulate those of ulcer, and one is unable to determine whether the condition is malignant at this stage. Nevertheless if differentiation is not possible we are able to determine when surgical interference should supplant medical treatment. When the Roentgen picture of the stomach shows us the presence of permanent defects in its shape, due to induration or adhesions, six hour rest, and abnormal peristalsis, it is then that surgery is indicated. At this early period it is possible often to produce a cure. W. J. Mayo, in a report of operations for Gastric Cancer, states that radical operation in this early stage has materially diminished the mortality. In the early stages peristalsis is very active, but the contents are expelled very slowly. A bulging just within the pylorus on the greater curvature occurs as the result of the contractions propelling the food towards the pylorus which is not patent, and the pre-pyloric portion stretches further to the right than the pylorus, causing the pylorus itself to assume a position practically on top of hte stomach and pointing towards the spleen. Early this bulging is slight and increases as the involvement progresses. Another cardinal symptom is the so-called filling defect. This is caused by the growth extending into the lumen of the stomach and causing an irregularity in its contour. In the medullary carcinoma the filling defects are larger and multiple, while in the scirrhus type they are smaller, the stomach assuming a funnel shape. Hour glass contractions may then result from the attack of the tumor mass. Filling defects caused by organic changes must be differentiated from those caused by other means, such as growths of neighboring organs or a colon distended by gas or by spasmodic contraction. Defects caused by a gas filled splenic flexure indenting the greater curvature are frequently met with. Fluoroscopically, palpation of the stomach away from the colon will clear up doubt concerning the cause of irregularity. Deformity caused by spasmodic contraction will disappear under atropine medication.

Abnormal peristalsis is commonly seen in Gastric Cancer. As the acidity of the stomach diminishes peristalsis in proportion becomes less marked. A peristaltic wave progresses as far as the mass, skips it and resumes its activity. Reverse peristalsis is a sign noted by some observers. The motility is diminished because of the the involvement of adjacent organs, producing fixation of the stomach. Residue is dependant upon the extent of the obstruction. If the pylorus is gaping there may be none at all.

### GASTRIC AND DUODENAL ULCER.

The Rontgen diagnosis of ulcer is of a comparatively recent date. In the early use of the opaque meal it was thought that ulcer could be diagnosed by the bismuth adhering to the lesion. It is now known that the hyper-motility produced by the irritated surface causes marked contractions which, if anything, propel the bismuth from the ulcer instead of causing it to adhere.

Gastric Ulcer occurs most frequently in the pylorus and pyloric region. Simple acute ulcers are as a rule cured by medication, and those which are not amenable to medication and become chronic are more frequently seen by the Roentgenologist. In chronic ulcer the inflammatory tissue after a time produces changes which in turn produce deformity. It is necessary to determine if any deformity or change in contour is due to an actual lesion or to a functional disturbance. Serial Radiography and Fluoroscopy will determine this. Any defect not altered by palpation or anti-spasmodics and remaining permanent after extended observation, is a direct sign of an organic lesion.

In penetrating ulcer the bismuth fleck is seen in a so-called pocket or diverticulum, the bismuth often remaining present for a great length of time. This sign was first called attention to by Haudek, and is known as the Haudek niche.

The indrawing of the circulature of the stomach known as the incisura is a frequently seen Rontgen sign. This is as a rule produced by an infiltration or irritation caused by the ulcer. Hour glass deformity is also of great diagnostic value.

Retention of a considerable portion of the bismuth meal for more than six hours in gastric ulcer is usually due to pylorospasm, or if in the area of the pylorus the residue is a result of the obstruction at that site from scar tissue.

### DUODENAL ULCER.

Duodenal ulcer is diagnosed with greater ease and certainty than gastric ulcer. George states that 95% of all duodenal ulcers occur in the first portion of the duodenum, and that if normal the first portion is always seen on the plate with its characteristic shape and smooth outline, that any constant deformity of the cap is indicative of a pathological condition which may be ulcer, adhesions due to cholecystitis, or accidental variation, such as pressure from adjacent organs. Carman, at the Mayo clinic, has divided the Roentgen signs of duodenal ulcer into two groups consisting of major and minor signs.

The major signs are:

1st. Gastric hyper-peristalsis.

2nd. A residue in the stomach (sometimes in the duodenum after six hours if there be obstruction from scar tissue contraction).

3rd. A diverticulum of perforating ulcer.

The minor signs are

1st. Gastric hyper-motility with early free opening of the pylorus and speedy clearing of the stomach.

2nd. Gastric hypertonus.

3rd. Irregularities in the outline of the cap or bulb of the duodenum.

4th. Lagging of bismuth in the duodenum.

5th. Pressure tender point over the duodenum.

6th. Spasm of the stomach, such as hour glass or slowly traveling incisura.

Case and Cole consider permanent defects in the duodenal cap one of the chief means of diagnosing duodenal ulcer and its complication. I have found in my own experience that when a series of plates reveals a constant permanent defect or deformity in the cap, a pathological condition exists in the duodenum itself or its adnexa.

The diagnosis of obstruction at the pylorus is made invariably by the X-ray. The usual causes are ulcer and malignancy occurring within the stomach and tumor masses from without causing pressure. Chronic appendicitis with adhesions involving the cecum, transverse colon and omentum, the latter drawn towards the appendix and making traction on the greater curvature interfere with the motility. Adhesions of the gall bladder and pylorus in cholelithiasis and cholecystitis may produce a narrowing of the pylorus and subsequent obstruction. Syphilis is a less common factor in the production of pyloric obstruction, but must be considered.

### GASTROPSIS.

Gastropsis is a condition very frequently encountered Roentgenologically. Under the screen the bismuth is seen to fall through the stomach very rapidly. The oesophagus with the fornix are held by their ligaments above, and the pylorus by its ligaments below, so that the bismuth distends the lower portion of the stomach, which subsequently increases in its vertical length. Above the walls of the stomach are in contact, the fornix containing gas. Peristalsis in the erect position is nearly absent, but is more pronounced in recumbency. If atony of the wall is also present, dilatation will occur unless compensatory muscular hypertrophy is present. In gastroptosis associated with atony the stomach may retain its contents for eight or more hours.

### CHOLELITHIASIS AND CHOLECYSTITIS.

The Rontgen diagnosis of gall stones is possible in a large number of cases. Many observers report their ability to detect the presence of stones in a large percentage of cases. A. W. George says that in 85-95% of cases he is able to demonstrate stones. Calculi composed of pure cholestrin cannot be shown, but those containing calcium even in small amounts are visible. It should be remembered that negative Rontgen findings do not rule out the presence of stones. Cholecystitis with adhesions to the pylorus is frequently diagnosed.

Often conditions in and about the appendix are discovered. As a rule the caecum fills in 6 hours, and the appendix soon after. If the appendix retains bismuth for 24 to 48 hours after the meal, according to Case, it is a diseased appendix in proportion to its defective drainage.

In the examination of the large bowel the Rontgen method is of great value. It is always advisable to employ the bismuth enema whenever any organic condition of the colon is suspected. From 3-5 ounces of the chemically pure barium sulphate are used in a suitable menstrum, and the solution permitted to enter by gravity.

The accepted Roentgen description of the bowel is as follows: "The caecum is that portion of the colon into which the ileum empties through the ileo cecal valve. It is almost surrounded by the peritoneum and is therefore freely movable. It may be found in the pelvis or displaced upwards.

The ascending colon extends upwards and backwards into the iliac fossa, and reaches nearly to the liver, where it forms a more or less acute angle. The hepatic flexure, together with the proximal portion of the transverse colon, may frequently be ptosed, drawn forwards and downwards. This is a normal condition for many people. The transverse colon extends from the hepatic flexure to the splenic flexure. It varies greatly in position. Particularly in thin individuals it may hang as a loop reaching into the pelvis. In this position the distal portion may appear to overlie the descending colon. The Roentgenoscope or stereoscopic plates are useful for differentiation in such conditions. The close relation between the transverse colon and the greater curvature of the stomach should be borne in mind.

The splenic flexure is firmly held to the diaphragm by a strong ileo colic ligament. This flexure normally occupies a position several inches higher than the hepatic flexure. The descending colon extends to the brim of the pelvis. This portion of the colon is practically retroperitoneal and is fixed. The sigmoid has a great normal variation in size and position and is attached by a mesentery which varies in length. The rectum extends from the external sphincter to the sigmoid. It is the most distensible portion of the colon, and consequently has a great normal variation in size and shape.

Following administration of the bismuth meal the various sections of the colon are reached as follows. The cecum between 4 and 6 hours, the hepatic flexure in 7 hours, the splenic flexure in 9 hours, and in 11 hours the column appears at the brim of the pelvis. It is difficult to discern peristalsis in the large bowel, the slow rythmic contractions not being visible. Holzknecht believes that the colon is not very active, except for intermittent contractions which propel the contents with sudden force. With the patient in the upright position sometimes the low position of the hepatic flexure causes constipation through kinking.

The cause of constipation may often be found by Roentgen examination. Hertz classifies constipation in two divisions, i. e.:

1st. Where there is delay in the passage through the colon.

2nd. Where the feces reach the sigmoid flexure in the normal time but are delayed in the rectum. He terms this inefficient defecation.

In cases of the first type, delay through the colon, the time consumed by the bismuth in approaching various parts of the colon may be noted. In the second type, that due to inefficient defecation, studies made before and after show shadows in the rectum, whereas if the rectum is clear after defecation, the constipation is due to the delay in the colon.

New growths are diagnosed by obstruction to the advance of the bismuth column. The clysma is of importance in these examinations, and in those cases where tumor formation is present the flow of the bismuth is interfered with. Dilatation of the colon on the proximal side of the tumor is also a sign. Case suggests that "At the 12th, the 28th, and possibly the 52nd and 76th hour the colon should be studied with special reference to the hindrance above the point already under suspicion."

PTOSIS.

Ptosis of any portion or of the entire colon may occur, and is easily recognized. The transverse colon may fall completely into the pelvis. Anomalies of the sigmoid are discernable, such as kinks, loops and aberrant sigmoid.

444 Franklin Street.

---

**Proposed Abolition of Heroin.** The Committee on Drug Addiction of the National Committee on Prisons, has developed the concensus of opinion that heroin is more used by juvenile criminals than other preparations of morphine and, since heroin is not absolutely indispensable but can be replaced with ordinary morphine salts. or even Galenicals of opium, federal legislation is proposed to prohibit its importation, manufacture and sale. Perfectly simple and logical. If people eat too much meat, and beef is found—as is highly probable—to be the most used of any, prohibit the raising of cattle. To check automobile accidents, count the cars, and close the factory that turns out the plurality—though it is claimed that it is now a majority. Instead of prohibition or restraint of alcoholic indulgence as such, pass a law that the most popular beverage shall be no longer made, etc., etc., etc.

---

**Blood Test.** H. Couturier, Med. Press, July 19, advocates the following: Haematoxylin in water, 5 parts in 10,000; sodium hydroxid, 40 5; hydrogen peroxid, 12 volumes. Add to 5 c.c. of the sodium hydroxid solution, shake and add a drops of haematoxylin solution. Make the test in duplicate, using water or preferably the same excretion that is to be examined but known to be free from blood. A dark blue tinge develops in both tubes. Add 10 drops of hydrogen peroxid and compare. If blood is present, this color changes to violet red in 3 or 4 seconds, to light brown in 20 seconds, and to pale yellow in 40 seconds, the same changes occurring in the control, but only after several minutes. The haematoxylin solution must be freshly made. (Interesting but apparently not so good as other tests.—Ed.)

